# The Effect of Ensiling on the Nutritional Composition and Fermentation Characteristics of Brown Seaweeds as a Ruminant Feed Ingredient

**DOI:** 10.3390/ani10061019

**Published:** 2020-06-11

**Authors:** Mairead Campbell, Jordi Ortuño, Lauren Ford, David R. Davies, Anastasios Koidis, Pamela J. Walsh, Katerina Theodoridou

**Affiliations:** 1 Institute for Global Food Security, Queen’s University Belfast, Belfast BT9 5DL, Northern Ireland, UK; mcampbell105@qub.ac.uk (M.C.); j.ortunocasanova@qub.ac.uk (J.O.); t.koidis@qub.ac.uk (A.K.); 2 School of Chemistry & Chemical Engineering, Queen’s University Belfast, Belfast BT9 5AG, Northern Ireland, UK; l.ford@imperial.ac.uk (L.F.); pamela.walsh@qub.ac.uk (P.J.W.); 3 Silage Solutions Ltd., Bwlch y Blaen, Ceredigion SY25 6DP, UK; dave.silage@gmail.com

**Keywords:** seaweed, silage, ruminants, alternative feed, fermentation, phlorotannins, in vitro dry matter digestibility, nuclear magnetic resonance

## Abstract

**Simple Summary:**

In recent years, there has emerged a renewed interest in the inclusion of seaweed as an animal feed ingredient. Due to annual fluctuations in the availability and biochemical composition of seaweeds, effective preservation methods are needed. These are currently restricted to thermal processing methods. Ensiling is a commonly applied preservation technique for terrestrial forages intended for livestock feed but little is known about the characteristics of silage made from seaweeds. This study considered the potential of ensiling two brown seaweed species (*Fucus vesiculosus* and *Saccharina latissimi*) with or without the use of a microbial inoculant. The potential applications of seaweed silage as a feed ingredient in ruminant diets were considered. The results showed that, depending on the species, ensiling may be a suitable preservation method for brown seaweeds.

**Abstract:**

Ensiling could be an effective method to preserve seaweeds for animal feed applications, however, there is limited scientific knowledge in this area. Seaweeds are a promising ruminant feed ingredient, in part due to the content of phenolic compounds, which are receiving considerable interest as alternative antimicrobial agents in feed. The aim of the study was to compare the effect of ensiling on the nutritional composition and fermentation characteristics of two brown seaweed species, *Fucus vesiculosus* (FV) and *Saccharina latissimi* (SL) with or without the use of a *Lactobacillus plantarum* (LAB) inoculant. The effect of ensiling on the stability of phlorotannin was also investigated using nuclear magnetic resonance (NMR). After harvesting, the seaweeds were wilted for 24 h and subsequently ensiled in laboratory-scaled silos for 90 days. SL silage showed a stronger fermentation pattern (pH < 4), dominated by lactic acid (50–60 g/kg Dry Matter (DM)), and a slightly higher acetic acid content compared to FV silages (*p* < 0.05). The fermentability of FV was limited (pH > 4.8) with low lactic acid production (<5 g/kg DM). The addition of the LAB inoculant showed no effect on the fermentation process but a modest effect on the chemical composition of both species was observed after the 90-day ensiling period. The results showed no losses in the nutrient content of FV after ensiling, however losses in the Crude Protein (CP, −32%), ash (−36%), Neutral Detergent Fibre (NDF, −77%) and Acid Detergent Fibre (ADF, −58%) content of SL were observed. The ensiling process had a limited effect on the in vitro true dry matter digestibility and phenolic content of either species. Therefore, ensilage may be a suitable preservation method for the use of brown seaweeds as a ruminant feed; however, species-specific differences were observed.

## 1. Introduction

Global seaweed production doubled from 14.7 in 2005 to 30.4 million tonnes in 2015 and continues to grow at an annual rate of about 6% [1]. Although the vast majority is currently destined for human consumption, either directly or indirectly (via the production of carrageenan, agar and alginates), seaweeds represent a versatile natural resource with multiple applications, including feeds, fertilisers, biofuels, cosmetics and medicines, amongst others [2]. Seaweeds were traditionally used as a valuable source of fodder for livestock diets in coastal regions [3]. In recent years, there has emerged a renewed interest in the use of seaweeds as an animal feed ingredient due to (i) their low reliance on land, freshwater and chemical inputs [2] (ii) their potential to reduce ruminal methane emissions when included in ruminant formulations [4] and (iii) their nutritional content in chelated minerals, complex carbohydrates, polyunsaturated fatty acids, protein and bioactive compounds, which may exert relevant biological activities [5]. Seaweeds can be divided into three types based on the pigmentation: brown (Phaeophyceae), red (Rhodophyceae) and green (Chlorophyceae). Brown seaweeds are the most studied and exploited type for animal feeding, given their wide availability, large mass yields and ease of harvest [6]. They also possess a wide range of bioactive compounds, including complex carbohydrates (alginates, sulphated fucose-containing polymers and laminarin), pigments (fucoxanthin) and phlorotannins [7]. The latter are a group of compounds which are exclusive to brown seaweeds. Phlorotannins are a class of phenolic compounds which have demonstrated similar antimicrobial properties compared to tannins found in terrestrial plants, but differ in terms of their structural composition [8]. Phenolic extracts from brown seaweeds exhibit a variety of biological activities when fed to animals, including antioxidant, anti-inflammatory and antimicrobial properties [9,10], and may even exhibit more potent biological activities compared to certain terrestrial tannins [11]. The biomass yield and nutritional composition of seaweeds are both highly variable over the growing season [12,13]. Therefore, feasible, post-harvest, preservation methods that guarantee a consistent year-round supply are needed [14]. After harvesting, seaweeds are commonly dried using natural or thermal methods which reduce the moisture content to <20%; this stabilizes the biomass for storage and transport [15]. Nevertheless, natural methods are strongly dependent on local production capacities and optimal weather conditions, whereas thermal methods are energy-demanding, particularly given the high moisture content of seaweeds (70–90%). Therefore, drying methods are impractical for large-scale seaweed processing [15]. The process of ensiling is a fermentation method that is widely applied to terrestrial livestock feeds. This method is particularly important in temperate regions and offers a less weather- and energy-dependent alternative to dried forages [16]. Current knowledge on the feasibility of ensiling seaweeds for animal feeding is limited. A recent study showed important species-related differences in the ensilability of seaweeds as a source of ruminant feed [17]. Other authors found limitations in the extent of a suitable lactic acid fermentation of seaweeds due to a high buffering capacity, the content of complex carbohydrates, which are resistant to microbial degradation, and poor natural lactic acid-producing microflora in fresh seaweeds [18,19]. Whilst the presence of phlorotannins has been proposed as a hindrance to fermentation processes through the direct suppression of microbial degradation [15,20], given their biological relevance in the ruminant diet, it is important to establish the effects of the ensiling process on the phlorotannin content of seaweeds. Within this context, it seems necessary to assess the ensilability of brown seaweeds, and to consider the addition of inoculants before storage to enhance silage quality. Microbial inoculants, namely *Lactobacillus plantarum* (LAB), are commercially available and have been successfully used for increasing the fermentation quality of the ensiled forage [21]. However, studies on the use of LAB-based inoculants on the quality of seaweed silage are limited and have been performed under different experimental conditions [17,22].

The aim of the study was to compare the effect of ensiling on the nutritional composition and fermentation characteristics of two brown seaweed species (*Fucus vesiculosus* and *Saccharina latissimi*) with or without the use of a LAB inoculant. The stability of phlorotannins, before and after ensiling, was also investigated to assess the potential of seaweed in ruminant nutrition as a functional feed ingredient.

## 2. Materials and Methods

### 2.1. Seaweed Sampling

Two species of brown seaweed, *Fucus vesiculosus* (FV) and *Saccharina latissimi* (SL) were randomly collected by hand from a sheltered beach in Bangor, County Down, Northern Ireland (N 54 39′, W 5 39′), either from the intertidal (FV) or subtidal area (SL), during low tide in July 2017. The areas were divided into three equal areas, and approximately 1-kg portions (fresh weight) of each species were collected from each area. Seaweed samples were identified by a marine phycologist and stored in sample bags to be transported to the laboratory. The seaweeds were washed thoroughly with cold fresh water to remove sand and particulate species prior to further processing. 

### 2.2. Laboratory-Scale Silage Preparation

The silage preparation was carried out according to the method of Wout et al. [23]. The washed seaweeds were spread thinly onto a large plastic sheet and wilted for twenty-four hours. For chemical analyses, samples of FV0 and SL0 of each species were immediately frozen at −20 °C, freeze-dried (Christ Alpha 2–4LD) and passed through a grinder fitted with a 1-mm sieve, to determine the chemical composition of the pre-ensiled material (FV0 and SL0). The remaining seaweeds were cut by hand into < 5 cm pieces and divided into 100 g (fresh weight, FW) sub-samples. Fifteen replicates per species were randomly selected for each treatment (inoculant/untreated) for a total of 60 mini silos. For the chemical composition of pre-ensiled material (FV0 and SL0), three sub-samples as replicates were randomly selected from the 60 mini silos. Prior to ensiling, the inoculated groups (SLi/FVi) were sprayed (1 mL/kg fresh matter) with a freshly prepared culture (1 × 10^9^ CFU/kg FW) of *Lactobacillus plantarum* (strain NCIMB 3014) previously dissolved in deionized water and thoroughly mixed by hand, according to the manufacturer’s instructions. The remaining seaweeds (SLu/FVu) were treated in the same way without the dissolution of the LAB culture in deionized water, and thus underwent natural fermentation. The seaweeds were vacuum-packed in sterile bags (dimensions 20 × 30 cm) using a vacuum packing machine (Buffalo CD969 Compact Chamber) to remove ≥ 95% of the air from the bags. The silos were then stored in the dark for 90 days. After the storage time, three bags from each treatment were randomly selected for fermentation analysis and the remaining bags were frozen at −20 °C, and later freeze-dried and milled through a 1-mm sieve for further chemical and in vitro digestibility analysis.

### 2.3. Silage Fermentation Analysis

Silage extracts (*n* = 3) were prepared by mixing 30 g of silage from each bag in 150 mL of deionized water. The pH of the filtrate was determined using a pH electrode (736 GP Titrino, Metrohm, Herisau, Switzerland) after shaking the solution at 4 °C overnight and filtering through Whatman No. 6 filter paper. Ammonia-N **(**NH_3_-N) was determined using a calibrated ammonia ion-selective electrode (Orion Research, 1990). Volatile fatty zcids (VFA) and alcohols were measured following ethanol/water extraction using gas chromatography analysis (Varian Star 3400 CX GC, Varian Inc., Palo Alto, CA, USA) equipped with a Zebron ZB-FFAP (Phenomenex, CA, USA) Capillary column (30 m × 0.53 mm i.d., 1.0 micron film thickness), and nitrogen as the carrier gas. The instrument was coupled to a flame ionization detector. 

### 2.4. Chemical Composition

Pre- and post-ensiled seaweed samples were analysed for dry matter (DM) (AOAC 930.15) and ash (AOAC 942.05) according to AOAC official methods [24]. Neutral detergent fibre (aNDF) was measured according to Van Soest et al. [25] and modified according to Mertens [26], using sodium sulphite and heat-stable α-amylase; results were expressed inclusive of ash. Acid detergent fibre (ADF) and lignin (sulphuric acid) were analysed according to AOAC [24] and Robertson and Van Soest [27], respectively. Nitrogen (N) content was analysed using a Leco Protein/NAnalyser (FP-528, Leco Corp., St Joseph, MI, USA) and crude protein (CP) was calculated using N×5.0, as suggested by Angell et al. [28]. The water soluble carbohydrate (WSC) content, expressed as glucose equivalents, was determined using the anthrone–sulphuric acid method, as described by McDonald and Henderson [29]. The mineral composition was measured using an energy dispersive X-ray fluorescence (XRF) spectrometer (Rigaku, NEX CG, Tokyo, Japan). *Porphyra umbilicalis* (NCS ZC73021), was used as certified reference material; a variability of ± 20% (of recovery) was considered acceptable. All chemical analyses were carried out in triplicate.

### 2.5. Determination and Stability of Phenolic Compounds

Pre- and post-ensiled seaweed samples were analysed for total phenolic content (TPC) using the Folin–Ciocalteu method, adapted from Li et al. [30]. Briefly, the seaweeds were extracted using 0.2 ± 0.05 g of freeze-dried seaweed material in an acetone–water mix (70:30; solid to liquid ratio 1:20). The mixture was ultra-sonicated in a water bath (USC600TH, VWR, International Ltd., USA) for 30 min at 20 °C and centrifuged for 2 min at 2200× *g* (Sorvall Legend RT, Hanau Germany). Following the addition of Folin– Ciocalteu (1N) reagent and aqueous sodium carbonate (20% *w*/*v*), the solution was stored in the dark for 60 min and the absorbance was read at 725 nm (Jenway 6305, Barloworld Scientific Ltd., Dunmow, Essex, UK). Phloroglucinol (Sigma-Aldrich, Dorset, UK) was used as an external standard (1.0 to 50 µg/mL), and results were expressed as g of phloroglucinol equivalents per kg DM (g PGE/kg DM). 

The stability of phenolic compounds was observed using nuclear magnetic resonance (NMR) by investigating the molecular structure of the compounds before and after ensiling. The number of scans was set at 256 and the chemical shifts were referenced by setting the TMS peak to 0 ppm. The seaweed extracts were prepared using 0.3 ± 0.05 g of freeze-dried seaweed material in a deuterated acetone–water mix (70:30; solid to liquid ratio 1:20). After 30 min at room temperature, the acetone was removed by rotary evaporation and the resultant solid was re-dissolved in 0.1 mL deuterium oxide (Sigma-Aldrich, Dorset, UK). ^13^C-NMR spectroscopy was carried out on a Bruker DPX600 NMR (Bruker, Germany) spectrometer. Chemical shifts were reported in parts per million (ppm).

### 2.6. In Vitro True Dry Matter Digestibility

In vitro true dry matter digestibility was determined using the Daisy^II^ incubation method, followed by NDF digestion in an ANKOM 200 Fibre Analyzer (Ankom Technology, Fairport, NY, USA), as described previously by Holden [31]. Ground and freeze-dried seaweed samples (0.5 ± 0.05 g) were weighed into triplicate bags (pore size, 50 µm), heat-sealed and placed in the digestion jars. Blank bags were also prepared to correct for bacterial contamination [31]. Prior to the digestion run, two buffer solutions (A and B) were prepared and warmed to 39 °C. The reagents used were: solution A (10 g of KH_2_PO_4_, 0.5 g of MgSO_4_·7H_2_O, 0.5 g of NaCl, 0.1 g of CaCl_2_·H_2_O and 0.5 g of urea in 1 L of deionized water) and solution B (15 g of Na_2_CO_3_ and 1 g of Na_2_S.9H_2_O in 1 L of deionized water). A final buffer solution was made by mixing solutions A and B (5:1 ratio) before adjusting the pH to 6.8. Rumen contents were collected from three post-slaughter bovine rumens from an abattoir in N. Ireland. The rumen contents were then isothermally transported to the laboratory, homogenised, filtered through four layers of cheesecloth and purged with CO_2_. The rumen fluid was added to the digestion jar containing the pre-warmed (39 °C) buffer solution (*v*/*v* 1:5). After a 48-h incubation period, the bags were rinsed four times with distilled water, and the residues were subjected to digestion for 60 min in neutral detergent solution and dried at 60 °C for 48 h. The in vitro true dry matter digestibility (IVTDMD) was calculated as the difference between the dry matter incubated and the residue after neutral detergent treatment divided by the dry matter incubated. A total of two in vitro incubation runs were carried out and each time the sample was incubated in triplicate.

### 2.7. Statistical Analysis

Statistical analysis was performed using JMP version 14.0 (SAS Institute Inc., Cary, NC, USA). The statistical model used for the analysis of variance (ANOVA) was:

Yij = μ + Si + Ij + (SI)ij + Eij(1)

where Yij is the dependent, continuous variable; μ is the overall mean; Si and Ij are the fixed effects of species and inoculant, respectively; (SI)ij is the interaction of effects and Eij was a residual error. Results were considered significant at 5% and were subsequently compared using a Tukey test. Principal component analysis (PCA) was performed using SIMCA 15.0 (Umetrics AB, Umeå, Sweden). The data were subject to auto-scaling, and the significance level for Hotelling’s T2 was set at 0.05.

## 3. Results

### 3.1. Seaweed Characteristics before Ensiling

**Figure 1:**
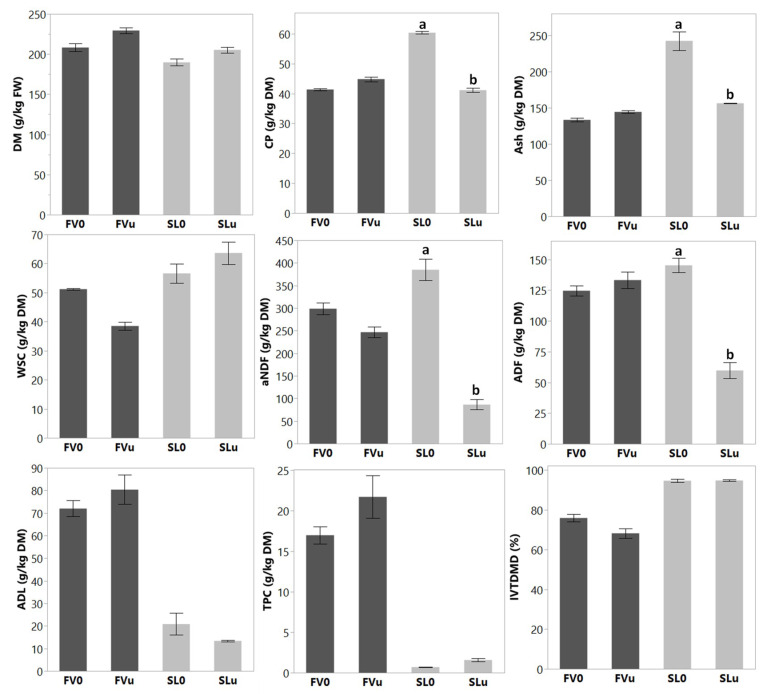
The chemical composition and IVTDMD of *Fucus vesiculosus* (FV: dark grey) and *Saccharina latissimi* (SL: light grey) before (0) and after ensiling (u: no inoculant). DM = dry matter; CP = crude protein; WSC = water soluble carbohydrate; aNDF = neutral detergent fibre; ADF = acid detergent fibre; ADL = acid detergent lignin; TPC = total phenolic content; IVTDMD = in vitro true dry matter digestibility. ^a,b^ Means within the same species with different lowercase letters are significantly different (*p* < 0.05).

**Figure 2:**
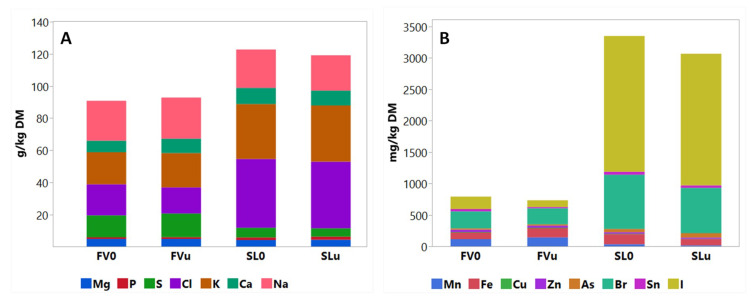
The macromineral (**A**) and trace element (**B**) composition of *Fucus vesiculosus* (FV) and *Saccharina latissimi* (SL) before (0) and after ensiling (u: no inoculant).

Figure 1 and Figure 2 illustrate the differences in the characteristics of the two species before ensiling (FV0 and SL0). Both species showed marked differences in all analysed parameters, except for WSC and some mineral components. The DM, TPC and ADL content of FV0 were higher (*p* < 0.05) compared to SL0. On the other hand, SL0 was characterized by higher (*p* < 0.01) CP, ash, aNDF and IVTDMD compared to FV0. In terms of the mineral composition (Figure 2), SL0 was exceptionally high in Cl (43 g/kg DM), I (2200 mg/kg DM), Br (862 mg/kg DM) and As (51 mg/kg DM), relative to FV0. FV0 was notably higher (*p* < 0.001) in Mn (114 mg/kg DM) and S (14 g/kg DM) compared to SL0 (34 mg/kg DM and 6 g/kg DM, respectively).

### 3.2. Seaweed Characteristics after Ensiling

**Figure 3:**
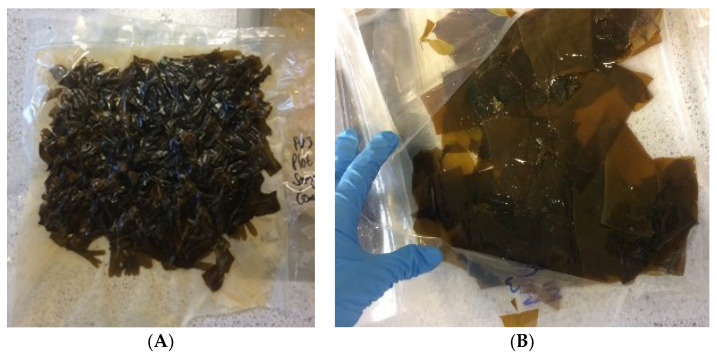
Seaweed silage ((**A**) *F. vesiculosus*, (**B**) *S. latissimi*) after a 90-day storage period.

Figure 1 and Figure 2 also show the effects of a 90-day ensiling period on the characteristics of the seaweeds. On initial visual inspection, all the samples appeared to be well preserved, with none or few signs of deterioration (Figure 3). After ensiling, the silage DM content of SL remained lower than that of FV. In contrast, the CP content showed no difference (*p* > 0.05) between the species, as the CP of SL reduced from 60 to 41 g/kg DM after 90 days. There was a large reduction in the aNDF and ADF content of SLu compared to SL0. Furthermore, the ash content of SL reduced from 242 to 156 g/kg DM after ensiling. Figure 2 shows the effect of ensiling on the mineral composition of the seaweeds. The mineral profiles changed in a similar way to the ash content in both species, with no variation in the most representative elements. However, a decrease (*p* < 0.05) in the following trace minerals were observed between SL0 and SLu samples, respectively: Mn (33 to 17 mg/kg DM), Fe (161 to 90 mg/kg DM), Zn (25 to 18 mg/kg DM) and Br (862 to 720 mg/kg DM). Ensiling had no effect on the high iodine content of SL, which remained >2000 g/kg DM after 90 days. Furthermore, the IVTDMD of SL remained high (>90%) after ensiling. In general, the ensiling process had a limited effect on the chemical composition or IVTDMD of FV; the differences observed among the species before and after ensiling seemed to be related to changes in the SL values. The addition of the inoculant showed a modest effect on the chemical composition of both species after the 90-day ensiling period (Table 1). The CP content of SLi seemed to be partially protected (*p* < 0.01) (45.7 vs. 41.1 g/kg DM), whereas a quantitatively modest increase (*p* < 0.05) in the WSC content was also observed for the same treatment. Regarding FV, FVi showed a higher (*p* < 0.05) TPC than FVu.

### 3.3. Silage Fermentation Characteristics

Table 2 shows the fermentation characteristics of the seaweeds after the 90-day ensiling period. The pH of FV silage ranged from 4.9 to 5.3, whereas the pH of SL silage was lower, ranging from 3.8 to 4.0. The high pH of FV silage was reflected in the low organic acid concentration (<6 g/kg DM) at the end of the storage period. In contrast, SL silage showed a stronger fermentation pattern, clearly dominated by lactic acid, ranging from 50–60 g/kg DM, and a slightly higher acetate content compared to FV silages (*p* < 0.05). Butyric acid and valeric acid were absent in all silage samples tested. The ethanol content was also higher in SL compared to FV silages. The use of the LAB inoculant showed no effect (*p* > 0.05) on any of the end-point silage fermentation parameters analysed. 

### 3.4. Determination and Stability of Phenolic Compounds

**Figure 4:**
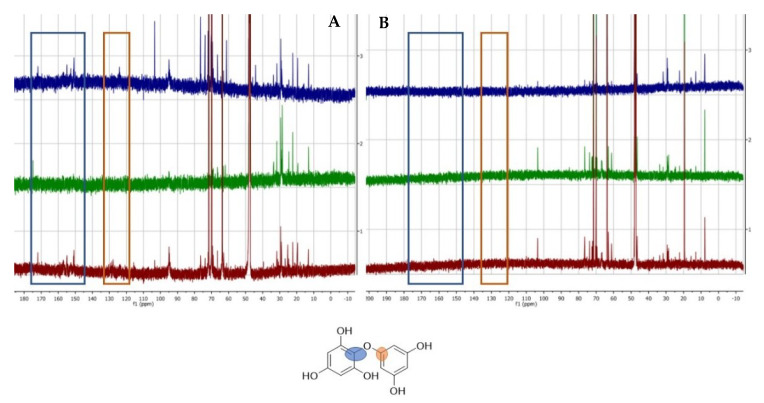
^13^C-NMR seaweed spectra of FV (**A**) and SL (**B**). The coloured spectra show the seaweeds before (FV0, SL0: blue) and after ensiling, with (FVi, SLi: green) and without (FVu, SLu: red) the inoculant. The blue and orange boxes represent the C-O-C bonds that are shown on the chemical structure below.

The TPC of FV0 was more than 20 times greater than that of SL0. Moreover, ensiling did not alter the TPC content of either species (*p* > 0.05; Figure 1). The ^13^C-NMR spectra displayed in Figure 4 shows the two characteristic areas corresponding to polyphenols according to the literature [8]. The areas between 120–135 ppm (orange box) and 150–170 ppm (blue box) are characteristic of polyphenolic compounds and phlorotannins. These characteristic peaks are highlighted in the example chemical structure in Figure 4, but the actual polyphenolic compounds can vary in size quite considerably. Analysis of these linkages by ^13^C-NMR in brown seaweeds has previously been reported by Ford et al. [32]. The spectra indicated a much stronger presence of polyphenols in FV (Figure 4A) than in SL (Figure 4B) seaweed samples, which was also reflected in the total phenolic content (FC) assays (Figure 1). The ^13^C-NMR spectra showed a reduced signal after ensiling FV, compared to FV0 (blue spectrum). This was particularly evident when the inoculant was applied to the ensiling process, which is also reflected in Table 1.

### 3.5. Principal Component Analysis

**Figure 5:**
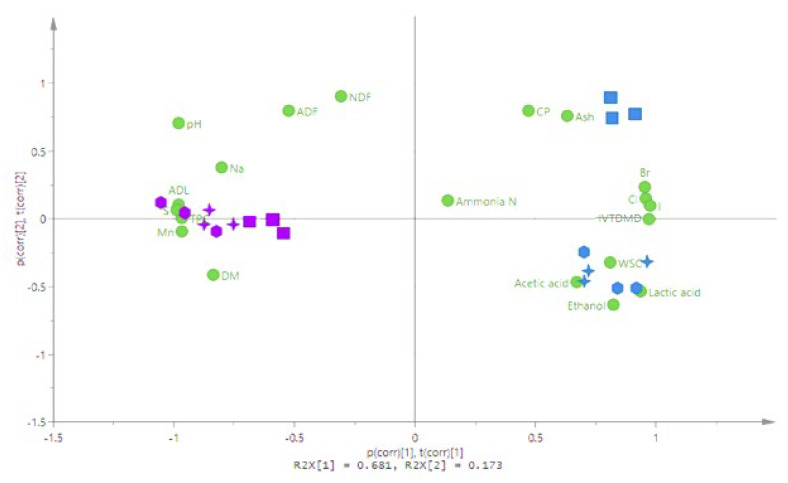
Biplot of principal component analysis showing the characteristics of *F. vesiculosus* (FV, purple) and *S. latissimi* (SL, blue) before and after ensiling, and with or without a *Lactobacillus plantarum* inoculant. Before ensiling = ⬛; after ensiling, untreated = ⎔; after ensiling, inoculated = ✢; ⬭ = FV and SL silages with and without inoculant; t(1)/(2) = Principal component 1 or 2. DM = dry matter; CP = crude protein; WSC = water soluble carbohydrate; aNDF = neutral detergent fibre; ADF = acid detergent fibre; ADL = acid detergent lignin; TPC = total phenolic content; IVTDMD = in vitro true dry matter digestibility.

The PCA highlighted the findings between the samples and treatments used in the current study (Figure 5). Focusing on the *S. latissimi* samples (Figure 5—blue colour), the analysis showed that the ensiled SL samples (either inoculated or untreated, SLi/SLu) were different from prior-to-ensiling samples (SL0). This is because ensiling inherently altered the composition of this species, as various chemical and physical parameters confirm. On the other hand, the same was not observed for the samples derived from the seaweeds of the *F.vesiculosus* species (FV0, FVi or FVu, Figure 5—purple colour). There was also no clear separation observed between the untreated and inoculated groups for either of the species, since their underlying chemical and physical parameters remained very similar. 

The biplot in Figure 5 also illustrates the correlations between the chemical composition, mineral profile and fermentation variables. Specific minerals (i.e., Na, Mn and S) were negatively associated (−0.80 to −0.99) with lactic acid and IVTDMD. The lactic acid content of the silages was negatively correlated with NDF (−0.98), ADF (−0.97) and TPC (−0.95).

## 4. Discussion

The current study found that the silage characteristics of brown seaweeds are species-dependent. The post-wilt DM content of both species remained lower (≤20% FW) than the recommended levels for optimal silage quality (>25% FW) [33]. A short wilt period (<24 h) was chosen to prevent seaweed deterioration, as some species are prone to microbial degradation and therefore must be processed promptly after harvesting [15]. SL showed a suitable pH reduction after the 90-day ensiling period, below that required for an effective fermentation process (< 4.2) for these values of DM (~ 20%) [33]. The accumulation of lactic acid in silages causes a rapid reduction in pH, halting further nutrient losses and the growth of spoilage microorganisms, such as enterobacteria and clostridia, which is evidenced by the absence of butyric and propionic acid [34]. The high lactic acid:acetic acid ratio (ca. 15:1) and the low production of secondary fermentation products (i.e., butyric acid), suggests that a homofermentative process was achieved by ensiling SL; this is an indicator of a good-quality silage [35]. Likewise, Herrmann et al. [18] and Cabrita et al. [17] found similar values for the final pH (<4) of SL silage and the predominance of lactic acid over low levels of butyric acid production. Alongside these findings and the results from this study, *Saccharina latissimi* is proposed as a good, ensilable substrate for lactic acid fermentation.

This is the first study to address the potential of ensiling FV for livestock feed. The fermentation of FV was limited compared to SL. The pH of FV silage failed to reach the optimal silage range (3.8– 4.2), as evidenced by a lack of organic acid production. These findings are in line with Herrmann et al. [18], who found that small amounts of fermentation products and a minimum pH of 5.4 described the characteristics of *Ascophyllum nodosum* silage. The limited lactic acid fermentation of FV was probably due to the high phenolic content. The TPC of FV0 was 17 g/kg DM, although contents of > 20 g/kg DM have been reported elsewhere [36,37]. SL is characterized by a lower TPC with typical values in the range of 0.7– 1 g/kg DM, which are in line with this study [36]. It is likely that variations in the chemical composition of seaweeds over the growing season will influence their ensilability. For example, summer is typically characterized by a higher TPC compared to winter [9,37,38]. Phlorotannins, which have been reported as the only phenolic compounds in *F. vesiculosus*, are produced as a defence mechanism, and as such have potent antimicrobial properties [39]. These may restrict the growth of lactic acid bacteria, in addition to spoilage microorganisms, as indicated by the lack of butyric and propionic acid in this study and in others [15,18]. The differences in the TPC between the two species likely explains the differences in the fermentation profiles. The results from this study confirm the limited fermentability of seaweeds rich in phenolic compounds.

The effects of any microbial inoculant on silage quality are dependent on a range of factors, including the original forage composition, epiphytic microflora, ensiling conditions and the type of inoculant used [40]. According to the results, the use of a *Lactobacillus plantarum* inoculant, at a rate of 10^9^ CFU/kg fresh seaweed, had a limited effect on the fermentation quality of both species. This demonstrates that the seaweeds ensiled under natural conditions underwent a similar fermentation process compared to those provided with a microbial stimulant. This provides further evidence that seaweeds harbor endogenous enzymes required for the hydrolysis of complex carbohydrates, as suggested by Sandbakken et al. [41]. *L. plantarum* is a slow lactic acid producer at pH > 5, and therefore the inability of the pH to drop below 4.9 in FV silage probably limited the effect of the inoculant on silage quality. Furthermore, the low initial WSC content of both species (< 60 g/kg DM), and the inability of *L. plantarum* to degrade algal cell walls, together with the high content of Cl, I and other minerals, might have limited the inoculant’s efficiency [35,42]. Regarding SL, the use of the inoculant had a positive effect on the CP content of SL silage. Although the inoculant had no effect on the end-point pH, the mitigation of proteolysis was likely due to a more rapid fall in silage pH during the early fermentation phase [43]. These findings contrast with Cabrita et al. [17], who found that treating SL with an *L. plantarum* inoculant reduced the CP content of SL silages. Overall, the results showed that a favourable lactic acid fermentation was achieved, even without the use of the microbial inoculant. Further work is needed to explore the changes that occur within the microbial communities during the ensilage of seaweeds and the potential beneficial effects of different inoculant types on silage quality.

The initial content and availability of WSC can be a limiting factor during the ensiling process [44]. In the current study, ensiling had no effect on the WSC of brown seaweeds (Figure 1). Herrmann et al. [18] found that, despite the low initial WSC content of the brown seaweed *Saccorhiza polysaccharides* (10 g/kg DM), fermentation still occurred. The authors concluded that this was probably due to the use of alternative microbial energy sources. In fact, in the present study, the ensiling process decreased the aNDF and ADF fractions of SL from 384 to 86 g/kg DM and from 145 to 60 g/kg DM, respectively. A similar finding was observed during the ensilage of the red seaweed, *Gracilaria vermiculophylla* [17]. The storage carbohydrates in brown seaweeds are mainly composed of laminarin and mannitol, whereas alginate represents the main structural component [12]. Laminarin and mannitol, despite not being included within the WSC fraction [35], represent up to 330 and 190 g/kg DM of SL, respectively [7], and are utilized as a substrate for lactic acid bacteria during the ensiling process [41]. The structural carbohydrates, i.e., alginates, are likely to be more resistant to microbial degradation in the silo [15]. Therefore, the losses in aNDF and ADF are likely due to the utilization of laminarin and/or mannitol during the ensiling process. Whilst the content of alginate undergoes little annual variation, mannitol and laminarin tend to accumulate during summer in SL and decline during winter, as they are utilized as an energy source [12,45]. The high content of fermentable storage carbohydrates in summer-harvested SL [45] might make this the most suitable period to harvest seaweeds for ensilage. The differences in the chemical profiles observed between the species explained the higher ensilability of SL in comparison with FV (Figure 5). Principle component analysis (PCA) has recently been used to better understand the factors relating to silage characteristics [46,47] and can be used to correlate silage fermentation traits, chemical composition and the digestibility of different feeds [48]. Our findings suggest that seaweeds characterized by low ADL and TPC had the highest digestibility and ensilable traits, which is in agreement with Herrmann et al. [18]. In addition to TPC, the results also suggest that a high content of specific minerals (S, Mn and Na) probably had an inhibiting effect on the ensiling process, as suggested by previous authors [15,49].

Moreover, ^13^C-NMR was used to assess the differences in the linkage profiles of the purified phenolic extracts before and after ensiling. The ^13^C-NMR spectra in Figure 4 present the two characteristic areas corresponding to polyphenols in seaweed [32]. The phlorotannin compounds are linked by C−C or C−O−C bonds. The nature of the linkages will affect the overall structure of the phlorotannin and possibly its reactivity. Little is known about the effect of the overall linkages present in the phenolic mixture and their biological relevance [32]. This is the first study to investigate and compare the linkage profiles of pre- and post-ensiled seaweeds and the stability of these linkages after the ensiling process. Regarding SL, it was concluded that due to the low phenolic content (< 2 g/kg DM), the concentrations of phlorotannins were too low to be observed by NMR, due to the lack of sensitivity of the technique. It was found that the phenolic compounds of FV, namely the ether type linkages (C−O−C), displayed a relatively similar pattern before and after ensilage, without the presence of the inoculant. Therefore, this form of linkage was preserved throughout the ensiling process. However, with the addition of the *L. plantarum* inoculant, the signals from the phenolic compounds were relatively diminished, which indicated that there was a degradation of the phenolic compounds. This is also reflected in the FC assay data, although it is noteworthy that the FC assay is indirect and less specific compared to the NMR method [38,50]. Nonetheless, due to the agreement of the two techniques, it is suggested that the preparation of seaweed silage using a microbial inoculant may cause some structural changes to occur within the phlorotannin compounds. Due to the complexity of these compounds, further elucidation would be required to fully appreciate the effect of ensiling and inoculation on the biological properties of the phenolic compounds in seaweeds. It is worth highlighting that these experiments showed that upon ensiling FV without inoculation, the ratios of the characteristic peaks of polyphenols are comparable to that of the fresh (i.e., pre-ensiled) seaweed. This suggests that the structures of phlorotannins remain intact during the ensiling process; a similar finding was observed in tannin-containing terrestrial silages [51]. Hence, we would expect seaweed silage to have a similar bioactivity compared to pre-ensiled seaweeds, but further studies would be needed to confirm this hypothesis.

In terrestrial-based forages, protein represents 75–90% of the total nitrogen content. After ensiling, much of the nitrogen is transformed into soluble non-protein nitrogen, which is rapidly degraded by microbes in the rumen, thus implying a loss in protein use efficiency. Ammonia-N is commonly used as an indicator for the extent of proteolytic activity during the ensiling process [35]. Previously, Carbita et al. [17] found that after a nine-week ensiling period, the NH_3_N content of SL silage was between 50 and 70 g/kg CP, which is in agreement with this study, which found the NH_3_N of FV and SL to be 53 g/kg CP and 63 g/kg CP, respectively. Whilst the ensiling process did not incur any losses in the CP content of FV, a decrease from 60 g/kg DM to <46 g/kg DM CP was observed for SL. This might be explained by the lower phenolic content of SL compared to FV. The protein-binding capacity of phenolic compounds in seaweeds has been demonstrated previously [10] which could have reduced the availability of FV proteins to proteolytic microorganisms in the silo. Despite the loss of protein after ensiling SL, there was no difference in the NH_3_N content between FV and SL silages. Instead, other products of protein degradation may have been generated [52], which were not measured in the current study.

The ash contents of FV0 (133 g/kg DM) and SL0 (242 g/kg DM) were comparable to levels reported previously for the same species [12,53]. The ensiling process had no effect on the ash content of FV, but a reduction of up to 35% was observed after ensiling SL, as shown by previous authors [18]. The SL mineral analysis showed few changes in the content of major elemental constituents, however, losses of some trace minerals (i.e., Mn, Fe, Zn and Br) were observed. This contrasts with previous findings, which found that ensilage could be a method of reducing the high salt content of brown seaweeds [14]. Despite these losses, the ash content of SL silage still exceeded levels in FV and those found in terrestrial forages [54]. Previous authors have warranted caution when considering the supplementation of livestock diets with seaweeds due to the high content of specific minerals, namely As, Pb and I, which limit their inclusion in the ruminant diet [55]. The Pb concentrations in both species (before and after ensiling) were below the required limit (10 mg/kg DM) for livestock feed, whilst SL exceeded the 40 mg/kg DM threshold for the arsenic content in seaweed meals, as indicated in the European Union Directive 2002/32/EC on undesirable substances in animal feed [56]. The current study found that ensiling had no effect on the content of these potentially harmful minerals. Both pre-ensiled and SL silage were also exceedingly high in iodine (>2000 mg/kg DM) compared to FV (<200 mg/kg DM). Previous authors noted incremental increases in blood and milk iodine content when the diet of dairy cattle was supplemented with *Ascophyllum nodosum*; this could have implications for milk safety from a human nutrition perspective [57]. Based on the iodine content of SL in this study, and on estimations by Cabrita et al. [55], the inclusion of SL silage in the ruminant diet would be limited to <6 g/100 g dry matter intake due to the high iodine content.

Previous studies have noted the differences in the digestibility of various seaweed species in the ruminant diet due to biochemical differences in their composition [6,58]. In agreement with this study, Greenwood et al. [58] also found higher in vitro dry matter digestibility of SL compared to FV (53 and 17 %), using rumen fluid from grass-fed sheep. These values are comparatively low compared to the current study, which could be due to the harvest period (April versus July). More recently, other authors have also noted lower values for SL silage <50% [17]. The seaweeds in the current study were harvested from wild seaweed populations with unknown growth stage, history or age; factors which may have influenced the characteristics of the seaweeds [59]. SL (commonly known as “sugar kelp”) is highly regarded for the high fermentable sugar content [18,59] which could partly explain the differences in the IVTDMD between FV and SL. Surprisingly, the losses in the aNDF and ADF content of SL during the ensiling period did not result in a reduction in IVTDMD. This is in contrast to Cabrita et al. [17], who observed the extensive hydrolysis of structural carbohydrates in *Gracilaria vermiculophylla* silages which negatively affected the dry matter digestibility. However, in the current study, the ensiling process had no effect on the IVTDMD of SL. The reason for this is unclear, but it suggests that seaweed components that were not measured by detergent fibre analysis were utilized by the rumen microbes during the in vitro incubation period. The digestibility of seaweeds is expected to be highly variable and dependent on the species, the carbohydrate profile and composition (protein, minerals, phlorotannins), as well as on the adaptation of the animal to the feed [6]. In vivo studies on the digestibility of seaweed silage are limited and are therefore required to fully elucidate its value in the ruminant diet. Furthermore, the high ethanol content of seaweed silage has been highlighted by previous authors [17,18] and has been attributed to the content of epiphytic, salt-tolerant yeast populations [60,61]. Indeed, SL is known to naturally undergo a mixed fermentation by lactic acid bacteria and yeast populations [60]. This was evident in the current study, as ethanol content was high in SL silages (16 g/kg DM), which is in line with Herrmann et al. [18]. Feeding silages with an elevated ethanol content (>10 g/kg DM) could influence the quality and organoleptic properties of milk when fed to dairy cows [62], which would have considerable practical implications for the use of seaweed silage as a feed source. Therefore, the effects of feeding seaweed silage on ruminant fermentation metabolism, and the consequences for product quality, need to be established.

## 5. Conclusions

The present study found species-related differences in the silage characteristics of brown seaweeds. SL silage showed a homofermentative pattern, with a suitable pH decrease dominated by lactic acid production. Whilst the TPC and IVTDMD of SL were unaffected by the ensiling process, losses in CP, ash, NDF and ADF were observed. In contrast, FV underwent a restricted lactic acid bacteria fermentation despite the use of an inoculant, with few changes in the chemical composition post-ensiling. The *Lactobacillus plantarum* inoculant had a minor effect on silage quality, although proteolysis was partially mitigated by its addition in SL silages. The high I content, particularly regarding SL, is a major concern for the use of seaweed silage as a ruminant feed. Finally, ensiling had a minor effect on the phlorotannin content of brown seaweeds but a better understanding of their biological activity post-ensiling is needed to improve our appreciation of their contribution to the nutritive value of seaweed silage. Further questions regarding optimal dietary inclusion rates and the potential effects on animal productivity, including milk and meat quality, need to be addressed before the use of seaweed silage as a ruminant feed can be implemented.

## Figures and Tables

**Table 1:** Chemical composition and IVTDMD of the seaweed silages, treated with (FVi, SLi) and without (FVu, SLu) a *Lactobacillus plantarum* inoculant, after 90 days (*n* = 3) (g/kg DM unless stated otherwise).

Chemical Profile	FVu	FVi	SLu	SLi	SEM	*p*		
						Species	Inoculant	Species * Inoculant
DM (g/kg FW)	229.2	226.6	205.1	202.2	4.46	<0.001	0.773	0.974
CP	44.8 ^a,b^	45.0 ^a^	41.1 ^b^	45.7 ^a^	0.94	0.130	0.009	0.029
Ash	144.4	136.8	156.2	151.0	5.06	0.046	0.524	0.828
WSC	38.5 ^c^	41.9 ^c^	63.6 ^b^	75.2 ^a^	2.86	<0.001	0.025	0.170
aNDF	247.6	236.4	86.4	86.4	10.5	<0.001	0.676	0.538
ADF	133.3	126.4	59.8	46.4	4.96	<0.001	0.476	0.242
ADL	80.4	83.4	13.4	13.4	4.66	<0.001	0.647	0.516
TPC	21.7 ^a^	15.1 ^b^	1.5 ^c^	1.3 ^c^	1.33	<0.001	0.024	0.043
IVTDMD (%)	68.1 ^b^	67.4 ^b^	94.7 ^a^	94.2 ^a^	1.63	<0.001	0.931	0.967

FV = *F. vesiculosus*; SL *= S. latissimi*; FW *=* fresh weight; DM = dry matter; CP = crude protein; WSC = water soluble carbohydrate; aNDF = neutral detergent fibre; ADF = acid detergent fibre; ADL = acid detergent lignin; TPC = total phenolic content; IVTDMD = in vitro true dry matter digestibility; Species * Inoculant indicates the interaction of effects. ^a,b,c^ Means with different superscripts are significantly different (*p* < 0.05); SEM = standard error of the mean.

**Table 2:** Fermentation characteristics of the seaweed silages, treated with (FVi, SLI) and without (FVu, SLu) a *Lactobacillus plantarum* inoculant, after 90 days (*n* = 3) (g/kg DM unless stated otherwise).

Fermentation profile	FVu	FVi	SLu	SLi	SEM	*p*		
						Species	Inoculant	Species * Inoculant
pH	4.9	5.0	3.9	3.9	0.09	<0.001	0.928	0.846
Lactic Acid	2.8	2.5	56.6	54.9	1.65	<0.001	0.767	0.751
Acetic Acid	2.5	3.1	3.9	3.7	0.42	0.024	0.600	0.382
L:A	0.9	0.6	14.7	15.1	1.05	<0.001	0.946	0.789
Propionic Acid	ND	ND	0.3	ND	0.10	0.159	0.143	0.158
n-Butyric Acid	ND	ND	ND	ND	-	ns	ns	ns
i-Valeric Acid	ND	ND	ND	ND	-	ns	ns	ns
Ethanol	4.0	6.1	18.1	13.9	2.33	<0.001	0.388	0.196
Propanol	ND	ND	ND	ND	-	ns	ns	ns
Ammonia N (g/kg CP)	56.8	50.0	61.2	64.9	10.01	0.348	0.862	0.606

Species * Inoculant indicates the interaction of effects. ND = not detected; L:A = lactic acid:acetic acid ratio. Means with different superscripts are significantly different (*p* < 0.05); ns = no significant difference; SEM = standard error of the mean.
